# Phototherapy to Facilitate Wound Healing Following Pacemaker Infection: A Promising Tool to Improve Outcomes

**DOI:** 10.19102/icrm.2024.15124

**Published:** 2024-12-15

**Authors:** James Kneller

**Affiliations:** 1 University of Arizona College of Medicine—Phoenix, Phoenix, AZ, USA

**Keywords:** Extraction, optogenetics, phototherapy, pocket infection, wound care

## Abstract

Device infection remains a dreaded and increasingly common complication of pacemaker procedures, often mandating removal of all implanted materials. Intensive wound management may be necessary following extraction, requiring multiple follow-up encounters in the outpatient setting. Here, a case of pacemaker pocket infection necessitating complete system extraction is presented. A cutaneous phototherapy device (X39^®^; LifeWave, Inc., San Diego, CA, USA) was used to facilitate wound closure. Healing was found to occur 40%–50% faster with this adjunctive therapy, reducing the number of follow-up visits by half. These adhesive patches contain natural compounds that reflect back infrared frequencies emitted by the skin. Biologic activity includes elevation of glycyl-L-histidyl-L-lysine levels, with a plethora of effects. This non-pharmacological wellness device may be useful to hasten wound healing and recovery from pocket infection.

## Introduction

The use of cardiovascular implantable electronic devices (CIEDs) has risen dramatically over the past decades. Rates of infected hardware have also increased, necessitating removal of all implanted materials.^1,2^ Following device extraction, wound-management strategies are decided on an individual basis. Healing by secondary intention, which is also known as granulation wound care, is a technique where the wound is left open to heal naturally. New tissue fills in from the bottom up as the sides close in. This approach is selected in up to 28% of surgical cases, typically when there is an ongoing risk of infection or significant tissue loss.^3^ The time for closure depends on the wound size, depth, and location. Larger wounds can take ≥16 weeks, even with proper care.^4^

Although common, there is a lack of robust evidence to guide effective management of open surgical wounds.^5^ Non-pharmacological modalities such as negative pressure therapy are widely used interventions.^3^ Open wounds are also managed with frequent dressing changes and packing of the wound cavity, which is typically performed by hospital or community nurses. Prolonged healing times are a burden for both patients and caregivers. Affordable and easily implemented technologies that shorten the healing time may be highly impactful for both patients and caregivers.

## Case presentation

**Figure 1:**
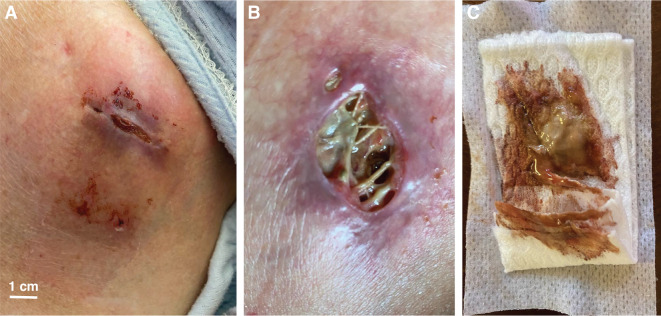
Recent pacemaker implant with poor early wound healing **(A)**, progressing to frank pocket infection **(B)** with purulent discharge **(C)**. See text for details.

**Figure 2:**
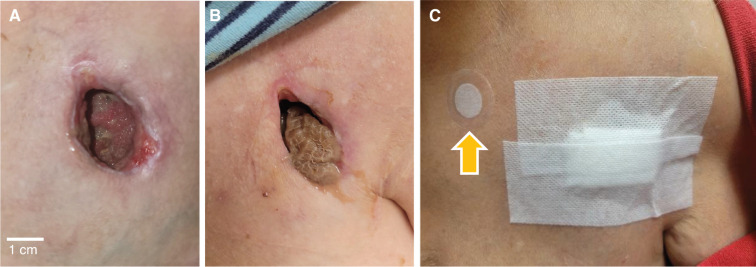
Evacuated pacemaker pocket following device extraction **(A)**. Would healing by secondary intention, with wound packing **(B)** and dressing changes **(C)** performed every other day until complete closure. Cutaneous phototherapy device (arrow) worn for 12 h daily to facilitate wound healing **(C)**. See text for details.

This case involves an 83-year-old woman referred for pacemaker pocket infection **(Figure 1)**. As she was not pacer-dependent, complete device removal was possible with no requirement for backup pacing. A complete capsulectomy was performed with debridement of involved tissues, leaving a substantial wound crater **(Figure 2A)**. Given the burden of tissue removed and concern for residual infection, healing by secondary intention was advised. The patient was discharged the day following device removal with an arrangement for outpatient wound care (FIRSTAT Home Health Services, Phoenix, AZ, USA). The wound protocol involved packing the cavity with silver alginate, which was reapplied every other day **(Figure 2B)** and covered with a gauze dressing **(Figure 2C)**. Five hundred milligrams of Keflex^®^ (cephalexin; Sandoz GmbH, Kundl, Austria) was given orally twice daily over the initial 4 weeks.

Given the patient’s desire for rapid recovery and preference for complimentary alternative medicine, a cutaneous phototherapy device (X39^®^; LifeWave, Inc., San Diego, CA, USA) was used to hasten wound healing **(Figure 2C)**. The X39^®^ adhesive skin patch increases serum concentrations of the human peptide glycyl-L-histidyl-L-lysine by >60%,^6^ which is known to accelerate skin regeneration and deep tissue repair.^7^ We hypothesized that this technology may hasten wound healing in the context of CIED procedures.

**Figure 3:**
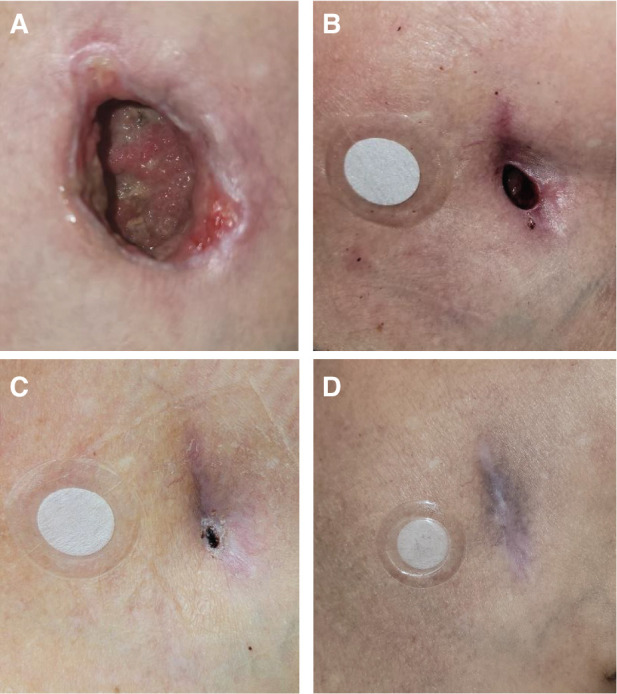
Progressive wound healing from the time of extraction **(A)**, after 6 weeks of standard wound care plus phototherapy. Granulation tissue is apparent at the base of the wound, indicating a robust healing response **(B)**. Scab formation was evident after 10 weeks, indicating complete closure **(C)**. Healing was estimated to be 50% faster with the use of phototherapy in addition to standard care. Phototherapy was continued, with progressive scar maturation evident at 16 weeks **(D)**. The outer diameter of the X39^®^ phototherapy patch is 34 mm. See text for details.

As per the manufacturer recommendations, patches were applied for 12 h daily **(Figure 3)**, with a 12-h patch-free interval overnight to avoid desensitization of the photobiomodulatory mechanism.^8,9^

The patches were placed adjacent to the wound and applied daily until wound healing was complete with minimal scarring. It was observed that wound closure with X39^®^ occurred 40%–50% faster relative to comparable cases managed by FIRSTAT. The patient also experienced ancillary benefits from phototherapy, including improved appetite, alertness, and less dependence on home oxygen therapy with X39^®^ as previously described.^10^

## Discussion

The emerging field of cardiac optogenetics uses targeted pulses of light to stimulate optically sensitive proteins in the heart, influencing cellular physiology with unprecedented spatiotemporal resolution.^11^ For example, low-intensity illumination can be used to terminate abnormal heart rhythms.^12^ Such technology harnesses the biologic activity of photons, but is limited by the need for external sources of light. In contrast, the LifeWave technology harnesses the light that is naturally emitted by the body to exert meaningful biologic effects.

Phototherapy with the X39^®^ system consists of an adhesive skin patch measuring 34 mm in diameter. The patch is non-transdermal, meaning no chemicals, drugs, or other substances are absorbed through the skin. The patches contain a proprietary blend of plant-based amino acids, water, stabilized oxygen, sugar, salt, and natural organic compounds.^13^ The human body spontaneously emits very weak light, typically in the visible or near-infrared spectrum, which is <1/1000 times the sensitivity of the naked eye. The photon emissions are thought to originate from the generation of free radicals during energetic metabolic processes. The patches stimulate the skin by reflecting back specific wavelengths of the emitted light, which are biologically active throughout the body.^13^

Molecular mechanisms include elevation of glycyl-L-histidyl-L-lysine levels,^6^ which is known to activate stem cells; increase collagen, elastin, and glycosaminoglycan synthesis; support the function of dermal fibroblasts; and stimulate blood vessel and nerve outgrowth while exerting powerful anti-inflammatory activity.^14^ The cardiovascular benefits of LifeWave phototherapy devices include improved heart rate variability and autonomic function,^13^ improved microvascular circulation, increased glutathione and carnosine levels, and lean body composition.^8^

### Limitations

This study presents a single case without a control group. The rates of wound healing are not standardized, and no assumptions can be made as to how quickly the pocket would have healed without this therapy. Wound vacuum–assisted closure would likely provide similar benefits. Larger studies are needed to generalize these results and compare the available supportive technologies.

## Conclusion

A wearable phototherapy technology was found to accelerate wound healing, thereby reducing morbidity while improving patient satisfaction. Light-based technologies in the emerging field of cardiovascular optogenetics may improve on these findings while introducing additional applications.
